# Thrombotic Microangiopathy After Kidney Transplantation: An Underdiagnosed and Potentially Reversible Entity

**DOI:** 10.3389/fmed.2021.642864

**Published:** 2021-04-08

**Authors:** Ana Ávila, Eva Gavela, Asunción Sancho

**Affiliations:** Nephrology Department, University Hospital Dr. Peset, Valencia, Spain

**Keywords:** thrombotic microangiopathy, kidney transplantation, atypical hemolytic uremic syndrome, eculizumab, complement system activation

## Abstract

Thrombotic microangiopathy is a rare but serious complication that affects kidney transplant recipients. It appears in 0.8–14% of transplanted patients and negatively affects graft and patient survival. It can appear in a systemic form, with hemolytic microangiopathic anemia, thrombocytopenia, and renal failure, or in a localized form, with progressive renal failure, proteinuria, or arterial hypertension. Post-transplant thrombotic microangiopathy is classified as recurrent atypical hemolytic uremic syndrome or *de novo* thrombotic microangiopathy. *De novo* thrombotic microangiopathy accounts for the majority of cases. Distinguishing between the 2 conditions can be difficult, given there is an overlap between them. Complement overactivation is the cornerstone of all post-transplant thrombotic microangiopathies, and has been demonstrated in the context of organ procurement, ischemia-reperfusion phenomena, immunosuppressive drugs, antibody-mediated rejection, viral infections, and post-transplant relapse of antiphospholipid antibody syndrome. Although treatment of the causative agents is usually the first line of treatment, this approach might not be sufficient. Plasma exchange typically resolves hematologic abnormalities but does not improve renal function. Complement blockade with eculizumab has been shown to be an effective therapy in post-transplant thrombotic microangiopathy, but it is necessary to define which patients can benefit from this therapy and when and how eculizumab should be used.

## Introduction

Thrombotic microangiopathy (TMA) is a life-threatening disease, characterized by endothelial dysfunction and the presence of thrombi in small blood vessels. As the thrombus forms, there is platelet consumption and mechanical disturbance of red blood cells, leading to thrombocytopenia and microangiopathic hemolytic anemia. Vessel occlusion results in tissue ischemia and organ damage, primarily affecting the kidneys, although other organs can be involved (1–4).

TMA syndromes can be classified according to the pathogenic mechanism (5, 6). Primary TMA syndromes include TMA whose etiology is known: thrombotic thrombocytopenic purpura (TTP), due to deficiency of the von Willebrand factor-cleaving protease ADAMTS13 (7); typical hemolytic uremic syndrome (HUS), caused by Shiga toxin-producing *Escherichia coli* (8); pneumococcal-associated HUS (9, 10); and atypical HUS (aHUS) caused by inherited or acquired abnormalities in complement proteins leading to unregulated activation of the alternative pathway of the complement system and the formation of the membrane attack complex (MAC) (11, 12). Other genetic causes, such as diacylglycerol kinase ε, an endothelial cell and podocyte protein, and cobalamin C deficiency have been described as causes of primary aHUS, mainly in children (13, 14). Secondary TMA syndromes occur in the context of infections, organ transplantation (solid organ and hematopoietic stem cell transplantation), drugs (cancer chemotherapy, vascular endothelial growth factor [VEGF] inhibitors, immunosuppressants such as calcineurin inhibitors [CNIs] and mammalian target of rapamycin inhibitors [mTORis]), malignancies, pregnancy, malignant hypertension, and autoimmune diseases (systemic lupus erythematosus, antiphospholipid syndrome, scleroderma, and vasculitis) (3, 5, 6). The distinction between primary and secondary TMAs is not absolute because genetic variants have been identified in patients with secondary TMAs. Moreover, secondary TMAs are also called secondary aHUS, because a complement deregulation has been described in some of those conditions, suggesting an overlap between these categories (15).

Post-transplant thrombotic microangiopathy (PT-TMA) is a rare but devastating condition that can lead to poor patient and graft outcomes. It can occur as a *de novo* disease or as a recurrence of a previous aHUS (sometimes undiagnosed before kidney transplantation). *De novo* PT-TMA is caused by various pathogenic mechanisms, whereas aHUS recurrence is a consequence of complement system deregulation triggered by several activating conditions (Table 1). The primary aHUS normally requires a second hit for disease to develop. The aHUS triggers match several situations that can produce secondary TMA, making the distinction between the two entities difficult (16).

**Table 1:** Causes of post-transplant thrombotic microangiopathy.

1. Caused by complement protein mutations: atypical hemolytic uremic syndrome
2. *De novo* post-transplant associated TMA or secondary aHUS a. Related to the type of donor and the organ procurement - Complement activation associated to DBD and CDC - Ischemia reperfusion injury b. Associated to post-transplant events - Drugs - Calcineurin inhibitors - mTOR inhibitors - ABMR - Infection - Viral: CMV, parvovirus, Nile fever - Funghi Antiphospholipid syndrome c. Other causes of TMA not related to the kidney transplant: malignancies, pregnancy, other drugs (anti VEGF, gemcitabine,…)

*DBD, donor after brain death; DCD, donor after cardiocirculatory death; ABMR, antibody mediated rejection; CMV, cytomegalovirus*.

## Epidemiology of Post-Transplant Thrombotic Microangiopathy

Post-transplant TMA is observed in 0.8–14% of kidney transplants (17, 18). (USRDS) *de novo* PT-TMA is much more frequent than recurrent aHUS (90 vs. 10% of all cases), but the risk associated with the development of PT-TMA is much higher (36.5 times; 29 vs. 0.8%) in patients with a history of aHUS (18).

## Clinical Manifestations of Post-Transplant Thrombotic Microangiopathy

The manifestations of PT-TMA are quite variable and can range from a limited form confined to the kidney to a full-blown systemic variant (19, 20, 24). The systemic form is typically acute, consisting of the classic triad of thrombocytopenia, microangiopathic hemolytic anemia with increase in lactate dehydrogenase, reduced haptoglobin, and schistocyte formation, and acute kidney injury (AKI); this has been described in 18–62% of patients with PT-TMA (19, 21, 25, 26). The localized form can manifest as isolated AKI or as a chronic form with slowly progressive graft dysfunction, proteinuria, or difficult-to-control arterial hypertension, and it can only be diagnosed when a kidney biopsy is performed (5, 27).

Although aHUS recurrence and PT-TMA are clinically and pathologically indistinguishable, a personal and family history of aHUS, an abrupt onset, and a complete and systemic TMA are suggestive of aHUS recurrence (28). Extrarenal manifestations of aHUS apart from hemolytic anemia (29–34) are frequent in aHUS recurrence, but they are rarely observed in *de novo* PT-TMA (35).

PT-TMA and aHUS recurrence can appear at any time in the post-transplant course (17, 36), but they develop primarily in the first 3 months after transplantation (21, 37), in conjunction with the presence of more complement activating events (e.g., ischemia-reperfnusion injury, high immunosuppressive drug levels, higher infectious risk). Systemic TMA usually appears in the early post-transplant period, and the localized form can appear at all stages after transplantation.

## Histological Changes

In the kidney biopsy, the active lesions are intraluminal fibrin occlusive thrombi along with endothelial cell activation signs, such as endothelial swelling, fragmented red blood cells in capillaries, mesangiolysis and microaneurisms, myocyte necrosis, and intramural fibrin in arterioli. Immunofluorescence microscopy is negative, except for fibrinogen. Electron microscopy shows subendothelial widening by flocculent material (“subendothelial fluff”). In the chronic phase, the characteristic lesions are double contour formation in peripheral walls with hyaline deposits in arterioles and fibrous intimal thickening with concentric lamination (onion skin). By electron microscopy, new subendothelial basement membrane and widening of the subendothelial zone can be observed (15, 21, 25, 38). The lack of thrombi in the biopsy does not exclude TMA (15). The biopsy does not allow the identification of the etiology, although some changes might suggest certain etiologies, such as C4d deposition, peritubular capilaritis, and glomerulitis in antibody mediated rejection (ABMR) or intimal thickening, reduplication of the elastic lamina, and hyaline degeneration in arterial hypertension. In PT-TMA, histological characteristics can be influenced by the donor's previous injuries. Thus, the interpretation of chronic injuries can be more complex. The findings of any acute injuries in the biopsy, together with clinical and laboratory data, can lead us to suspect TMA activity and the need for active treatment.

After the diagnosis of TMA, the etiology of the native kidney ESRD should be investigated to rule out previously missed aHUS (38).

## Prognosis for Post-Transplant Thrombotic Microangiopathy

The overall prognosis for PT-TMA is quite poor for the allograft and for the patient, resulting in a graft loss rate of 33–40% in the first 2 years (17, 18, 20, 21, 23), and a patient survival of 50% at 3 years after TMA diagnosis in a previous series (18); recently, however, 97% 1-year patient survival has been reported (25). Recurrence of aHUS appeared in 60% of transplant patients with the disease, leading to 89–90% graft loss in the first year in the pre-eculizumab era (39). In case of PT-TMA, although poorer short-term graft survival had been described in patients with the systemic form of PT-TMA, reflecting a more severe disease with a higher incidence of dialysis-dependent AKI and plasma exchange (PE) needs (19), the prognosis in the long term is similar in both forms (19, 25, 26). The prognosis is also similar in early (<3 months) and late (>3 months after transplantation) presentation (25).

## Causes of Post-Transplant Thrombotic Microangiopathy

At the time of kidney transplantation, the coincidence of several mechanisms that activate the complement system can trigger the recurrence of aHUS in patients with a genetic background or the development of *de novo* PT-TMA.

### aHUS Recurrence

In aHUS, the recurrence risk is determined by a genetic mutation in complement proteins (11, 15, 39–41, 48). Stratifying the risk according to the mutation is the key to making decisions about the post-transplant management of aHUS. A higher recurrence risk is considered in patients with recurrence in previous transplants and in carriers of pathogenic variants in CFH/CFB/CFH:CFHR1 rearrangements/TBHD; moderate risk in carriers of CFI variants/C3/anti-FH antibodies/homozygous for haplotypes CFH-H3/absence of variants (29, 39–48, 114); and low risk in those with isolated MCP/DGKE variants and with negative anti-FH antibodies at the time of transplantation (13, 40, 48–50) (Table 2). The use of prophylactic eculizumab reduced the post-transplant recurrence rate from 49 to 12% in patients with aHUS, reducing the probability of graft loss, and significantly increasing the number of patients with aHUS who receive a kidney transplant (51).

**Table 2:** Recurrence risk of aHUS after kidney transplantation in the preeculizumab era.

High (RR 80–90%)	- Previous recurrence - Pathogenic mutations in CFH - CFH/CFHR1 hybrid genes. >80 of recurrence rate. High graft loss risk (45, 114) - CFB > 80% of recurrence risk - TBHD = 80% (46, 47)
Moderate (RR 40–75%)	- Isolated CFI mutations. 40–60% recurrence risk (11, 39, 41, 44) - C3. 30–70%^*^ - Detectable circulating Anti–CFH antibodies (48–50) - Two at risk CFH haplotypes (39) - Negative complement genetic study 30% (11) - Complement gene mutation of unknown significance
Low (RR <20%)	- Isolated MCP mutation^+^(41) - DGKε mutation (13) - Negative Anti–CFH antibodies at the time of transplantation (48–50)

* *Recurrence risk varies depending on the different series*.

+ *Recurrences in patients with MCP account for patient with combined mutation/risk polimorphisms*.

*RR, recurrence risk; CFH, complement factor H; CFHR1 complement factor H related protein 1; CFB, complement factor B; TBHD, thrombomodulin; CFI, complement factor I; MCP, membrane cofactor protein; DGKε, diacylglycerol kinase epsilon*.

### Secondary Thrombotic Microangiopathy

In the absence of previous aHUS, Chua et al. (52) showed activation of the classical and terminal complement in several transplant-associated conditions: in donors after brain death (DBD) or donors after circulatory death (DCD) (53–55), or associated with ischemia reperfusion injury (56), immunosuppressive drugs (mainly CNI or mTORi) (41, 42, 57–61), ABMR (62–68), viral and fungal infection (69–82), and recurrence of antiphospholipid syndrome (APS) (83–85). The laboratory tests needed to evaluate causes of PT-TMA are described in Table 3. *De novo* aHUS has also been described in patients with C3 glomerulopathy in the native kidneys (86, 87) and in 1 patient with TTP (88).

**Table 3:** Main laboratory tests to perform in PT-TMA.

**Identify TMA**	Anemia Thrombocytopenia Schystocytes Elevated LDH Descended haptoglobin	- Dropping hematocrit - ≥25% drop from baseline - Often absent. Fairly pathognomonic when present - ≥2 fold increase
	Rising creatinine (or absence of improvement in recent transplanted patients) Proteinuria	- 25% increase or slow and progressive increase in sCr - >300 mg/d
**Rule out**	**TTP** ADAMTS13 activity <10% Plasmic score^*^	
	**STEC-HUS** Stool culture for *E. coli*	
	PCR in stool sample for shiga toxin	
	**CblC deficiency** Elevated plasma homocysteine Eleveated methylmalonic acid in urine or plasma Abnormalities in *MMACHC* gene	
**PT-TMA**	**DITMA** Immunosuppresive (CsA, TAC, EVE, SRL) plasmatic levels	
	**Infections** Blood PCR for CMV, BK virus Microbiological tests to discard Influenza, H1N1, HH6, HIV, HCV, Funghi	
	**ABMR** Antibodies to Class I + II HLA (Luminex)	

* *Plasmic score: a laboratory-derived scoring system to predict TTP. Five independent markers were identified as highly predictive of TTP, including a platelet count less than 30 × 10^∧^9/L, serum creatinine level less than 2.0, INR less than 1.5, mean corpuscular volume (MCV) less than 90, and the presence of a hemolysis variable (reticulocyte count greater than 2.5 percent, undetectable haptoglobin, or indirect bilirubin greater than 2.0 mg/dL). Absence of active cancer, solid-organ transplant, or stem-cell transplant have high negative predictability and were also included in the score. Each of the seven factors is given a score of one point if present. Score = 0 predicts 4.3 risk of TTP. Score = 7 predicts 96.2 percent risk of TTP. An intermediate score of five to six has limited utility with a TTP risk prediction of 56.8 percent*.

All these triggers appear in patients with or without a known genetic background; Le Quintrec reported 30% complement genetic mutations in a series of patients with *de novo* PT-TMA (29), indicating an overlap between recurrent and *de novo* TMA.

### Complement Activation Related to the Donor and Procurement Process

Activation of the complement system can be observed from earlier stages of transplant. This activation can be related to the type of donor. Naesens et al. (53) showed a relevant increase in expression of complement factors, such as C1, C3, and CFB, in renal allograft pre-implantation and after transplantation biopsies in DBD compared with living donor (LD) biopsies. Ischemia reperfusion damage has been associated with early injury of the renal allograft. Experimental data have suggested that, after ischemia reperfusion, complement is activated by the lectin pathway, and afterward, the alternative complement pathway could amplify the injury through the release of C3, C5, and the MAC. In addition, the damage to endothelial glycocalyx secondary to ischemia would reduce the union of factor H to the endothelial cells. In a similar manner, this complement activation after ischemia reperfusion would be higher in recipients of kidneys from DBDs than from LDs (54–56). The use of kidneys from DCDs has been associated with a higher risk of PT-TMA. The prolonged warm ischemia in this type of donor would aggravate the endothelial lesions in the graft and would increase complement activation and secondary damage (41, 112).

### Drug-Induced Thrombotic Microangiopathy

Drug induced TMA (DITMA) is suspected when there is a sudden onset acute kidney injury, usually within hours or a few days after drug exposure, and resolution can be observed when the drug is stopped or reduced (57, 89, 113).

The association between CNIs and *de novo* PT-TMA has been well documented in the literature, with the risk higher with cyclosporine than with tacrolimus (41). Various mechanisms have been associated with the development of PT-TMA after CNI treatment. The loss of normal equilibrium between vasoactive peptides, with an increase in vasoconstrictor substances, such as angiotensin II, thromboxane A2, and endothelin, and a reduction of vasodilatory molecules, such as prostaglandin (PG) E2, prostacyclin (PGI2), and nitric oxide leads to arteriolar vasoconstriction and endothelial injury secondary to renal ischemia. CNIs also favor platelet aggregation and plasminogen activation, with a higher risk of thrombosis. Cyclosporine causes endothelial cells to release microparticles that activate the alternate complement pathway (58).

The diagnosis of CNI-related TMA is found in the early post-transplant period when the levels of these drugs are high. Recently, new advances in the understanding of TMA and its association with complement abnormalities have questioned the relevance of CNIs in this disease. It has been suggested that there must be some predisposing factors for PT-TMA development in patients taking CNIs, given that more than 95% of renal transplant recipients receive this treatment. Data from USRDS have shown a higher PT-TMA incidence in patients without CNI treatment. In addition, results from a French aHUS registry have not shown the association between CNIs and PT-TMA, and the lack of CNI use did not prevent the recurrence of aHUS in this study group (42). It was thought that mTORi could be a good alternative to CNIs for patients with aHUS. Unfortunately, various studies have not shown this protective effect. Data from USRSD showed a higher incidence of PT-TMA with sirolimus than with CNIs, and the French registry results highlighted a higher risk of recurrence post-transplant in patients with aHUS when mTORis were used. However, the fact that mTORis might have been used as a rescue therapy after diagnosis of PT-TMA limits the interpretation of these results (18, 41, 57). Inhibition of mTOR inhibition leads to the death of endothelial progenitor cells and the decrease in renal expression of vascular endothelial growth factor (VEGF), which also would lead to a reduction in Factor H synthesis. Other factors, such as an increased procoagulant and a reduced fibrinolytic state, are also believed to contribute to the pathogenesis of TMA in patients taking mTORis (59, 60). Recently, the combined use of CNIs with mTORis is increasing as an alternative treatment for renal transplant recipients, but this combination increases the risk of PT-TMA compared with single medication treatment, particularly when the blood levels of both drugs are high (22, 23, 61).

### Antibody-Mediated Rejection-Associated Thrombotic Microangiopathy

The complement system plays an important role in ABMR. The donor-specific antibodies bind to human leukocyte antigens on the allograft endothelium and activate the classical complement pathway through C1q, leading to activation of C4 and C3. The deposit of C3b on the membrane of endothelial cells triggers the activation of the alternate complement pathway with the generation of the MAC, which produces cell lysis and an inflammatory infiltration (62). The histological finding of TMA in patients with ABMR has wide variability, between 4 and 46%, most likely as a consequence of the focal presentation of the disease (63, 64). A negative impact of TMA on ABMR has been described. Wu et al. (65) found lower graft survival in patients with ABMR and TMA compared with ABMR without TMA. Diagnosis of TMA in the sensitized kidney transplant recipient is predominantly confirmed by histological findings.

### Infection-Related Thrombotic Microangiopathy

Viral infections can trigger PT-TMA due to the endothelial trophism of the virus, which induces the expression of adhesion molecules and the release of von Willebrand factor, causing platelet adhesion and microvascular thrombosis (71). CMV is the most frequently involved virus (69–73). In all CMV-related TMA cases, treatment with intravenous ganciclovir and plasma exchange (PE) resolved hemolysis; however, a TMA recurrence occurred in one case, which was resolved with valganciclovir and eculizumab (71). Parvovirus (74–76), hepatitis C virus, and its treatment (77–79) and fungal infections such as histoplasmosis PT-TMA have also been described (80, 81). Recently a case of ABMR and TMA associated with Nile Fever has been reported (82).

### Other Causes of Post-transplant Thrombotic Microangiopathy

APS can cause ESRD due to large and small kidney vessel thrombosis and TMA. These symptoms can recur after transplantation, and eculizumab has shown a beneficial effect in patients with this disease, preventing and treating the recurrence of APS (83–85).

## Treatment of Post-transplant Thrombotic Microangiopathy

### Treatment of aHUS Recurrence

Classically, transplantation of patients with aHUS has shown low graft survival with a high rate of loss due to recurrence of the disease. Prophylactic treatment or recurrence treatment are the two alternatives. Prophylactic treatment based on plasmapheresis does not effectively prevent recurrence in patients with high or moderate risk mutations, and subclinical complement activation have been reported in these patients. Excellent results with eculizumab as the first-line therapy have been reported; the usefulness of a first dose 1 h before reperfusion and a second dose 24 h after transplantation has been considered, both to reduce secondary complement activation due to ischemia-reperfusion. In the treatment of recurrence, little success has been achieved after plasmapheresis; nevertheless, good results have been observed with an early introduction of eculizumab after recurrence (42).

### Treatment of *de novo* Post-transplant Thrombotic Microangiopathy

Treatment of *de novo* PT-TMA should be based on correcting the potential cause of the disease and varies depending on the time of onset. Given the extreme heterogenicity of the mechanisms related to the appearance of TMA, therapeutic maneuvers must be individualized. The first step is to avoid complement over-activation before donation, preventing renal hypoperfusion during organ procurement, and reducing cold ischemia time.

In cases of PT-TMA secondary to immunosuppressive medications, the first step is to reduce or stop the offending agent, by switching from a CNI to another CNI or to an mTORi. This approach can resolve the TMA, but the effectiveness of this strategy is controversial. Satoskar showed no difference in outcomes between changing immunosuppression or not (20). Belatacept, a cytotoxic T lymphocyte antigen 4-immunoglobulin fusion protein that inhibits T cell function, allows the minimization or discontinuation of endothelial toxic immunosuppressants such as CNIs and mTORis (90, 91). However, a higher risk of acute kidney transplant rejection compared with current standard immunosuppressive therapy has been observed after conversion to belatacept in kidney transplant rejection (92).

In ABMR-associated TMA the mainstay of treatment is plasmapheresis (PP), with or without IVIg and additional immunosuppression (65–68). Despite the implication of complement activation in ABMR, the efficacy of complement inhibitors for the prophylaxis or treatment of ABMR is difficult to assess with current clinical data and is yet to be established. Instead, eculizumab use is currently recommended as a rescue therapy in AMR-associated TMA when hemolysis persists despite maximal management including plasma exchange (PLEX) and in those with PLEX dependency (66–68).

PT-TMA that is unresponsive to the previous measures has typically been treated with PE. PE has been shown to reduce mortality in TTP patients (93, 94), and it was the first-line therapy for aHUS in the pre-eculizumab era. PE can remove vasoconstrictor molecules such as thromboxane A2 and mutant complement proteins and provides deficient factors such as PGI2-stimulating factor and normally functioning complement components (27, 36). Due to these effects in primary TMA, the use of PE was extrapolated to PT-TMA. In 2003, Karthikeyan et al. (36) reported a graft salvage rate of 80% with PE in addition to CNI withdrawal in 29 patients with biopsy-proven TMA. Epperla et al. (95) showed a 100% response rate in 5 patients using withdrawal of the suspected offending agent associated with PE in 4, eculizumab in 2, and rituximab in 1 patient. However, the use of PE in a large proportion of patients does not improve kidney function despite correcting the hematological abnormalities, and it is associated with a 20–42% risk of graft loss (97, 98). Schwimmer showed similar graft outcomes in a series of 742 PT-TMA transplants, regardless of whether they had received PE (19).

**Figure 1:**
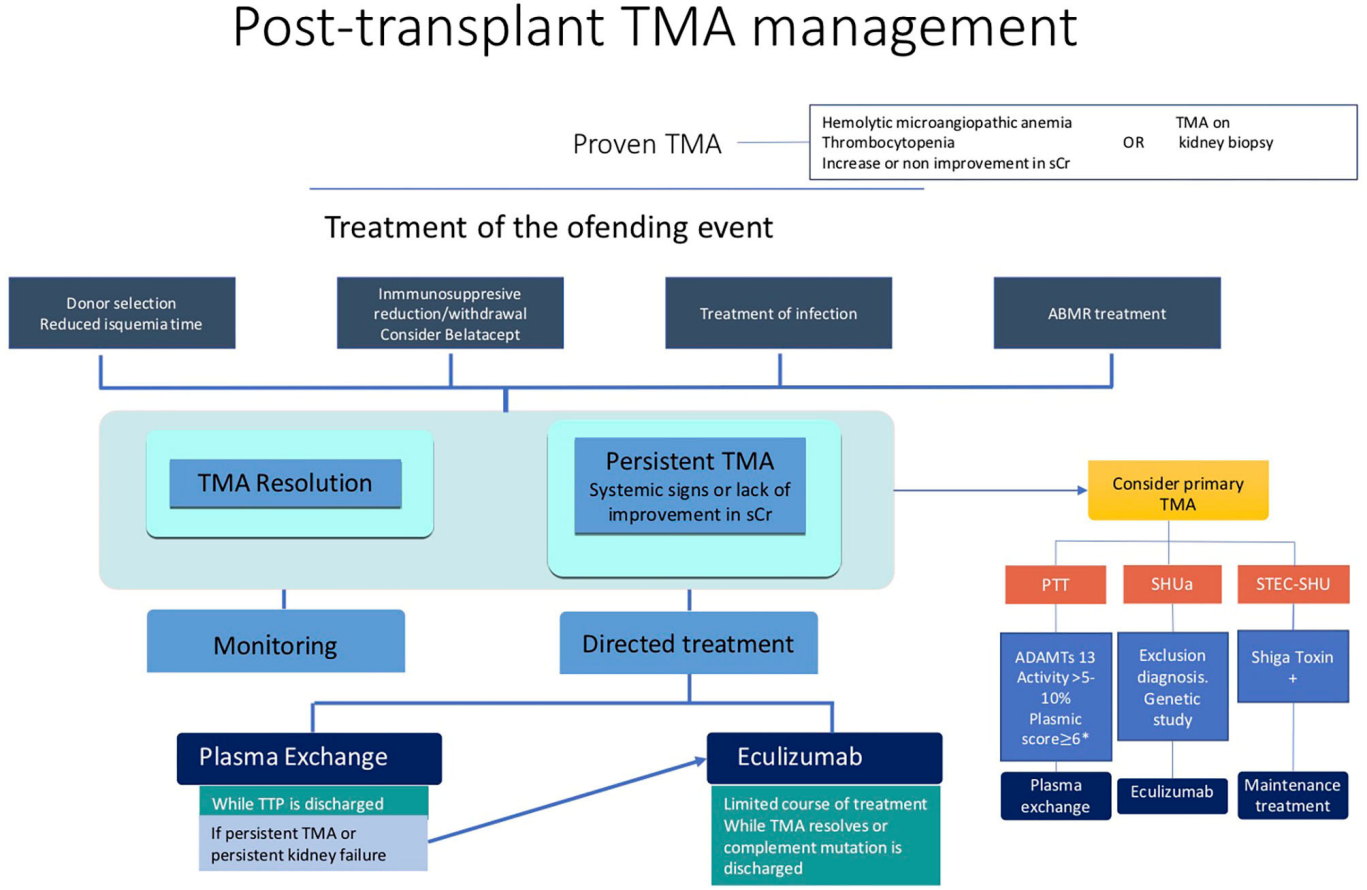
Post-transplant TMA management.

Eculizumab, a recombinant, fully humanized monoclonal antibody targeted against human complement protein C5, blocks generation of the lytic C5b-9 MAC. It has been shown to be effective in the treatment and prevention of recurrent aHUS after transplantation (99–103). In post-transplant TMA, a complement over-activation has been demonstrated (51–55, 104), even in patients without pathogenic complement proteins variants. Therefore, the inhibition of the complement system could be a suitable PT-TMA approach (Figure 1) (96).

The main risk associated with the eculizumab use is meningococcal infection; thus, vaccination against *Neisseria meningitidis* serogroups B, A, C, W135, and Y is mandatory. Vaccination should be administered 2 weeks before initiation of eculizumab treatment. But in PT TMA, usually it is not possible to delay eculizumab treatment two weeks, and prophylactic antibiotic should be used in the meantime. Given neither vaccines nor prophylaxis guarantee full protection against meningitis, patients and their families should be taught to recognize the alarm signs and symptoms of the infection (48).

Several case reports and small case series have been published documenting the efficacy of eculizumab in *de novo* PT-TMA refractory to previously mentioned treatments (Table 4) (53–61, 71, 83–85, 97, 98). Recently, the efficacy of eculizumab has been described in larger series. Cavero et al. (97) showed 15 kidney transplanted patients with PT-TMA; 14 patients were using tacrolimus, 4 of them combined with an mTORi. All but one withdrew the offending drug. Twelve patients also received PE (a mean of 5.46 sessions per patient, range 2–12) without improvements in serum creatinine (mean 4 mg/dl, 3.4–5.6). Eculizumab was started from 4 to 53 days after TMA detection, and a mean of 6.4 doses from 2 to 17 per patient were used. At the end of follow-up, mean serum creatinine was 1.7 mg/dl, with no graft losses or recurrences of TMA after eculizumab discontinuation. Portolés et al. (98) reported 22 patients with PT-TMA, 16 with early- (<1 month) and 6 with late- (>1 month post-transplant) onset TMA. All patients presented hematological TMA and most had a confirmatory biopsy. Some 77% of early and 100% of late patients with PT-TMA received PE, achieving a complete hematological response, but with an incomplete or an absent renal response in most cases. Eculizumab was then added, for an average of 21 days in the early TMA group and 83.5 days in the late TMA group. In the early group, 8 complete and 2 partial responses were observed, whereas in the late group, one patient had a complete response, two had a partial renal response, and the remaining patients lost their grafts. The patients with better responses had a shorter time lapse between diagnosis and the beginning of the treatment. Eculizumab was withdrawn in all cases, without relapses.

**Table 4:** Eculizumab in kidney transplant associated TMA^*^.

**Cause**	***n***	**Previous management**	**Eculizumab duration**	**Outcomes after eculizumab**	**Comments**
CAPS (83, 85)	8	Usual CAPS treatment	5w-Indefinite	3 pt: no CAPS relapse 5 pt: TMA resolution	CAPS prophylaxis in KT TMA treatment
Early CA (donor factors, Drugs) (53–61)	10	Treatment of the offending event PE	2 w−4 m^**^	7 pt: renal recovery and TMA resolution 1 pt: PRR 2 pt: graft loss	In all patients: more than one causal event 2 patients shared the same donor, indicating pre-tx CA
CMV (71)	1	Antiviral	1 yr	TMA resolution	Eculizumab+Valganciclovir
PT-TMA (97)	15	DW, DR PE 12 pt	2-52 w	TMA resolution Kidney function improvement	No graft loss No recurrence after ecu discontinuation
MAT PT (98)	16 Early 6 Late	DW,DR PE 13/16 pt PE 6/6 pt	3 w 11 w	8/11: CRR 2/11: PRR 1/6: CRR 2/6: PRR 3/6: Graft Loss	A shorter interval between TMA diagnosis and eculizumab leads to a better kidney function recovery

* *Antibody mediated rejection related TMA is not included*.

** *In one patient, the length of treatment is not described*.

*CAPS, Catastrophic antiphospholipid syndrome; Pt, patients; KT, kidney transplant; CA, complement activation; PE, plasma exchange; CRR, complete renal remission, PRR, partial renal remission; DW, drug withdrawal; DR, drug reduction*.

Eculizumab has been used more frequently in cases of lack of renal recovery after a short course of PE, obtaining an improvement of kidney function with few adverse events.

The duration of eculizumab treatment in cases of aHUS recurrence can be lifelong (depending on the genetic mutation); in secondary TMA, however, it is not clearly addressed. In previous reports, a short course of eculizumab was shown to be an efficient therapy to control TMA and to reduce the risk of graft loss due to this disease. Discontinuation of the therapy can be considered when complications of TMA have completely resolved, and on a case-by-case basis. It can also be considered when the kidney function has not improved after 3 to 6 months or when a lack of viability of the kidney graft is documented by means of a kidney biopsy or imaging tests (computed tomography, magnetic resonance imaging).

The current data are not strong enough to expand the recommendation for the use of eculizumab in all patients with PT-TMA, but in cases resistant to treatment of the offending event, eculizumab is a good therapeutic option. Although PE has been used, its efficacy is limited and it is important to note that in patients with PT-TMA, the sooner eculizumab starts, the better the renal function will be at the end of the follow-up (97, 98).

## Conclusion

The incidence and the impact of PT-TMA, either *de novo* or recurrent, on allograft survival is underestimated. Several factors related to the donor and procurement process and to the recipient and events during the post-transplant period (immunosuppressive drugs, rejection, and infections), trigger the development of this disease. The treatment of PT-TMA includes management of the offending event, PE, and recently, eculizumab, with promising results. However, further studies are needed to establish which PT-TMA kidney transplant recipients are most likely to benefit from eculizumab therapy, including when and how to use it given the high economic burden associated with this approach.

## Author Contributions

All authors contributed to the article and approved the submitted version.

## Conflict of Interest

AA have received fees for conferences from Alexion pharmaceutical. The remaining authors declare that the research was conducted in the absence of any commercial or financial relationships that could be construed as a potential conflict of interest.
